# Acute Kidney Injury Classified by Serum Creatinine and Urine Output in Critically Ill Cancer Patients

**DOI:** 10.1155/2016/6805169

**Published:** 2016-10-10

**Authors:** Bertha M. Córdova-Sánchez, Ángel Herrera-Gómez, Silvio A. Ñamendys-Silva

**Affiliations:** ^1^Department of Critical Care Medicine, Instituto Nacional de Cancerología, Mexico City, Mexico; ^2^Department of Critical Care Medicine, Fundación Clínica Médica Sur and Instituto Nacional de Ciencias Médicas y Nutrición Salvador Zubirán, Mexico City, Mexico

## Abstract

Acute kidney injury (AKI) is common in critically ill patients and is associated with higher mortality. Cancer patients are at an increased risk of AKI. Our objective was to determine the incidence of AKI in our critically ill cancer patients, using the criteria of serum creatinine (SCr) and urine output (UO) proposed by the Kidney Disease: Improving Global Outcomes (KDIGO).* Methods.* We performed a retrospective cohort analysis of a prospectively collected database at the intensive care unit (ICU) of the Instituto Nacional de Cancerología from January 2013 to March 2015.* Results.* We classified AKI according to the KDIGO definition. We included 389 patients; using the SCr criterion, 192 (49.4%) had AKI; using the UO criterion, 219 (56.3%) had AKI. Using both criteria, we diagnosed AKI in 69.4% of patients. All stages were independently associated with six-month mortality; stage 1 HR was 2.04 (95% CI 1.14–3.68, *p* = 0.017), stage 2 HR was 2.73 (95% CI 1.53–4.88, *p* = 0.001), and stage 3 HR was 4.5 (95% CI 2.25–8.02, *p* < 0.001). Patients who fulfilled both criteria had a higher mortality compared with patients who fulfilled just one criterion (HR 3.56, 95% CI 2.03–6.24, *p* < 0.001).* Conclusion.* We diagnosed AKI in 69.4% of patients. All AKI stages were associated with higher risk of death at six months, even for patients who fulfilled just one AKI criterion.

## 1. Introduction

Acute kidney injury (AKI) is very common in critically ill patients with a rate between 22 and 67% [1]. AKI severity is associated with increased mortality [2–4], a prolonged hospital stay, and high costs [5, 6].

Cancer patients are at an increased risk of AKI [7]. In addition to the common causes of AKI, cancer patients have risk factors associated with cancer or its treatment [8, 9]. The development of AKI prevents an optimal delivery of chemotherapy and is associated with lower complete remission rates [10, 11].

AKI is a frequent complication in critically ill cancer patients (CICP) with solid and hematologic malignancies [12, 13]. However, previous studies assessing AKI in CICP have used heterogeneous definitions of AKI. It was not until 10 years ago that studies began to include the criteria proposed by the Acute Kidney Injury Network (AKIN) and Kidney Disease: Improving Global Outcomes (KDIGO) to classify AKI based on the serum creatinine (SCr) and urine output (UO) [14].

Studies using the KDIGO criteria have reported an almost 70% incidence of AKI in critically ill patients with hematologic malignancies [15].

The objective of this study was to determine the incidence of AKI in our population of CICP using the criteria of SCr and UO proposed by the KDIGO. Our hypothesis was that the development of AKI correlates with higher mortality at six months, even for those patients with lesser degrees of AKI and for those who only have decreased urine output without elevated serum creatinine.

## 2. Materials and Methods

We performed a retrospective cohort analysis of a prospectively collected database of critically ill cancer patients. The institutional review board approved the observational study with the record Rev/012/16.

The Instituto Nacional de Cancerología (INCan) is a 180-bed public tertiary care cancer center, with a medical-surgical intensive care unit (ICU). We include all the consecutive patients admitted to the ICU from January 2013 to March 2015. We registered death during ICU stay and at six months.

We included patients above 18 years old requiring medical care for more than 24 hours in the ICU. In the case of multiple admissions, we considered only the first admission. We excluded patients with end-stage renal disease (ESRD) requiring renal replacement therapy or with incomplete clinical data.

At ICU entry, we collected gender, age, body mass index (BMI), hospitalization days prior to ICU admission, source, and cause of admission. The estimated glomerular filtration rate (eGFR) was calculated using the CKD-EPI (Chronic Kidney Disease Epidemiology Collaboration) equation [16]. The chronic kidney disease (CKD) was defined as an estimated glomerular filtration rate (eGFR) <60 mL/min/1.73 m^2^ for at least three months prior to admission [16]. Acute Physiology on Chronic Health Evaluation (APACHE) II score was calculated from the worst value obtained within the 24 hours of admission [17]. The Sequential Organ failure Assessment (SOFA) score was calculated at admission as an estimate of organ dysfunction [18].

We collected comorbidities, type of cancer and its extension, previous chemotherapeutic treatment, and Eastern Cooperative Oncology Group (ECOG) scale [19]. Leukopenia was defined as less than 4000 white blood cells per microliter.

During the ICU stay, vasopressor and mechanical ventilation requirements were collected. Urine output was assessed every 2 hours, total fluid balance was assessed every six hours, and serum creatinine was assessed every 24 hours. The main outcome was 6-month mortality.

### 2.1. AKI Diagnostic Criteria

AKI was defined as stated by the KDIGO clinical practice guidelines [14]. According to these criteria, grade 1 AKI is defined as >0.3 mg/dL SCr elevation within 48 hours or an increase in SCr from 1.5 to 1.9 times the baseline value SCr and/or UO < 0.5 mL/kg per hour for 6 to 12 hours; grade 2 AKI is defined as SCr elevation from 2 to 2.9 times the baseline value and/or UO < 0.5 mL/kg per hour for 12 to 24 hours; grade 3 AKI is defined as SCr elevation more than 3 times the baseline value or SCr over 4 mg/dL and/or UO < 0.3 mL/kg per hour for more than 24 hours, anuria during >12 hours, or requirement for renal replacement therapy (RRT).

We compared the criteria of serum creatinine and urine output. First, we classified patients according to their AKI stage by SCr elevation alone and then by the decrease in UO. In a third analysis, we classified AKI according to the worst grade reached by both criteria. In the case of multiple AKI episodes, we considered only the most severe episode.

We defined the baseline as the average SCr value in the three months preceding hospitalization. In patients without historical values, the baseline was calculated by estimating the eGFR at 75 mL/min/1.73 m^2^, as has been recommended [20].

### 2.2. Statistical Methods

We presented continuous variables as the means and standard deviations or the medians and 25–75% interquartile ranges. We expressed categorical variables as proportions. We compared AKI stages classified as either SCr or UO using analysis of variance (ANOVA) or Kruskal-Wallis test for continuous variables, according to their distribution, and we used chi-squared tests for categorical variables (Table 1).

We performed a univariate Cox-regression analysis of factors associated with 6-month mortality and selected variables with *p* values less than 0.05 for multivariate Cox proportional hazard models to estimate the magnitude of the associations. We constructed different multivariate models, to compare AKI classified by the criteria of SCr, UO, and the worst grade reached by both criteria.

APACHE II and SOFA scores were not used for multivariate analysis because they include renal function parameters and resulted in interactions; therefore, organ failure related variables were introduced separately. The data were analyzed using SPSS 22.0 (SPSS Systat Inc., Chicago, IL, USA) and Graph Pad Prism Version 5.01 software.

## 3. Results

Across the study period, of 447 patients admitted to the ICU, 48 did not have an adequate register of urine output for our analysis, nine patients spent less than 24 hours in the ICU, and one patient had chronic renal replacement therapy.

The remaining 389 patients fulfilled the inclusion criteria; 180 patients (46.3%) were male with a median age of 50 years (IQR 35–61). The median length of the hospital stay prior to ICU entry was 2 days (range IQR 1–5). Patients came from the operating room (184, 47.3%), hospital ward (183, 47%), and emergency department (22, 5.7%). The primary reasons for admission were sepsis or septic shock in 113 patients (29%), postsurgical care in 108 patients (27.7%), respiratory failure in 73 patients (19%), hypovolemic shock in 66 patients (17%), cardiac failure in 9 patients (2.2%), postreanimation care in 7 patients (1.8%), neurologic care in 5 patients (1.3%), and other reasons in 8 patients (2%).

Two hundred and eighty patients (72%) had solid tumors, and 109 patients (28%) had hematological malignancies. The most frequent types of malignancies were gynecological in 57 patients (14.7%), gastrointestinal in 53 patients (13.6%), acute leukemia in 45 patients (11.6%), non-Hodgkin's lymphoma in 38 patients (9.8%), soft tissue and bone sarcoma in 36 patients (9.3%), germinal in 31 patients (7.9%), breast in 20 patients (5.1%), and others in 109 patients (28%). Table 1 shows the main characteristics of the patients.

### 3.1. Acute Kidney Injury

The median baseline SCr was 0.75 mg/dL (IQR 0.6–0.94) with an eGFR of 100 mL/min/1.73 m^2^ (IQR 80.5–117).

When we classified AKI only by SCr, 192 patients (49.4%) had AKI, of which 73 (38%) had stage 1 AKI, 48 (25%) had stage 2, and 71 (37%) had stage 3. Classifying only by UO, 219 patients (56.3%) had AKI, of which 96 (43.8%) had stage 1 AKI, 72 (32.9%) had stage 2, and 51 (23.3%) had stage 3.

We classified patients according to their worst stage of AKI reached, either by SCr or by UO, and we identified 270 (69.4%) with AKI, of which 101 (37.4%) had stage 1 AKI, 84 (31.1%) had stage 2, and 85 (31.5%) had stage 3. Five patients with stage 3 AKI (5.9%) received intermittent hemodialysis. Patients with dialytic criteria, which had an end of life decision order or rejected treatment, did not receive RRT.

### 3.2. Outcome Analysis

The overall ICU survival was 77.6%, and the 6-month survival was 58.3%.

On the univariate Cox-regression analysis, we found the resulting factors associated with six-month mortality to be male gender (HR 1.64, 95% CI 1.2–2.25, *p* = 0.002), hospital days prior to ICU (HR 1.02, 95% CI 1.01–1.04, *p* = 0.004), APACHE II score (HR 1.06, 95% CI 1.04–1.08, *p* < 0.001), SOFA score (HR 1.2, 95% CI 1.16–1.24, *p* < 0.0001), sepsis (HR 1.83 95% CI 1.33–2.52, *p* < 0.001), leukopenia (HR 2.73, 95% CI 1.95–3.84, *p* < 0.0001), lactate (HR 1.07, 95% CI 1.01–1.13, *p* = 0.015), hematologic malignancy (HR 2.53, 95% CI 1.84–3.46, *p* < 0.001), neoplasm extension (HR 1.45, 95% CI 1.06–1.98, *p* = 0.020), ECOG (HR 1.28, 95% CI 1.11–1.46, *p* < 0.001), mechanical ventilation (HR 3.26, 95% CI 2.18–4.89, *p* < 0.001), and vasopressor use (HR 3.06, 95% CI 2.1–4.46, *p* < 0.001).

On the univariate Cox-regression analysis, the AKI stages classified by the different criteria that were associated with six-month mortality were serum creatinine alone (stage 1, HR 1.99, 95% CI 1.28–3.12; stage 2, HR 2.98, 95% CI 1.88–4.73; and stage 3, HR 4.05 95% CI 2.71–6.03, *p* < 0.001); urine output alone (stage 1, HR 2.11, 95% CI 1.35–3.29; stage 2, HR 3.11, 95% CI 1.98–4.89; stage 3, HR 7.45, 95% CI 4.76–11.67, *p* < 0.001); and worst stage reached by both criteria (stage 1, HR 2.84, 95% CI 1.61–4.98; stage 2, HR 4.38, 95% CI 2.52–7.6; stage 3, HR 8.44, 95% CI 8.44, 95% CI 4.96–14.36, *p* < 0.001).

We constructed different Cox-multivariate models using the AKI criteria (SCr, UO, and both), male gender, age, hospital days prior to ICU, sepsis, leukopenia, hematologic malignancy, neoplasm extension, ECOG scale, mechanical ventilation, and vasopressor use.

In the first multivariate model, we included AKI classified only by SCr, where the three stages of AKI were independent risk factors for mortality as follows: stage 1 with HR 1.7 (95% CI 1.07–2.70, *p* = 0.025), stage 2 with HR 1.98 (95% CI 1.22–3.21, *p* < 0.006), and stage 3 with HR 2.33 (95% CI 1.50–3.61, *p* < 0.001).

In the second multivariate model, we replaced SCr for the UO criteria. We found that only stage 2 and stage 3 were independently associated with mortality, but with a higher mortality risk than was associated with SCr elevation alone, stage 2 with HR 2.19 (95% CI 1.36–3.54, *p* = 0.001) and stage 3 with HR 4.18 (95% CI 2.52–6.92, *p* < 0.001).

**Figure 1:**
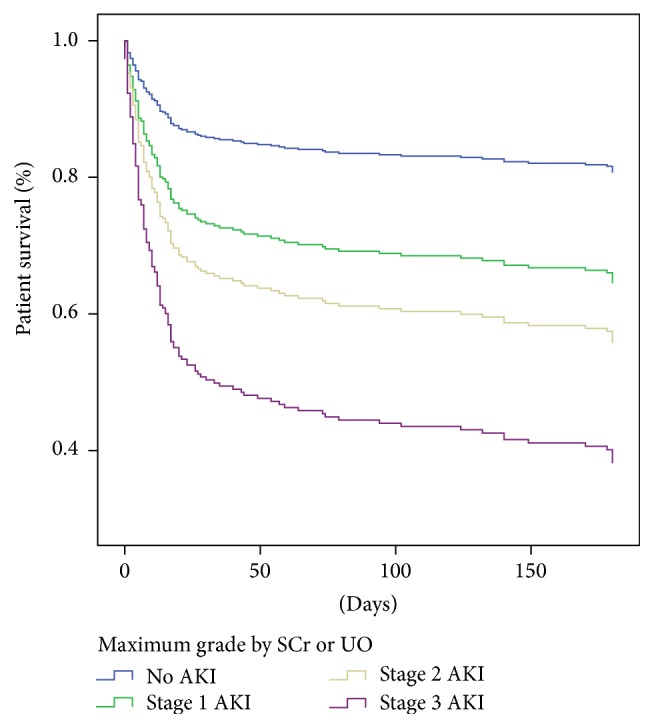
Cox proportional regression analysis curves, classifying patients by their maximum AKI stage, fulfilled by SCr or UO. Six-month mortality hazard ratios were HR 2.04 (95% CI 1.14–3.68, *p* = 0.017) for stage 1, HR 2.73 (95% CI 1.53–4.88, *p* = 0.001) for stage 2, and HR 4.5 (95% CI 2.52–8.02, *p* < 0.001) for stage 3.

Considering the worst grade reached, either by SCr or by UO, we found that the three criteria were independent predictors of mortality as follows: stage 1 AKI with HR 2.04 (95% CI 1.14–3.68, *p* = 0.017), stage 2 with HR 2.73 (95% CI 1.53–4.88, *p* = 0.001), and stage 3 AKI with HR 4.5 (95% CI 2.52–8.02, *p* < 0.001).  Figure 1 shows the mortality curves of the multivariate Cox proportional hazards regression analysis, classifying patients by their maximum AKI stage.

### 3.3. One or Two Criteria

**Figure 2:**
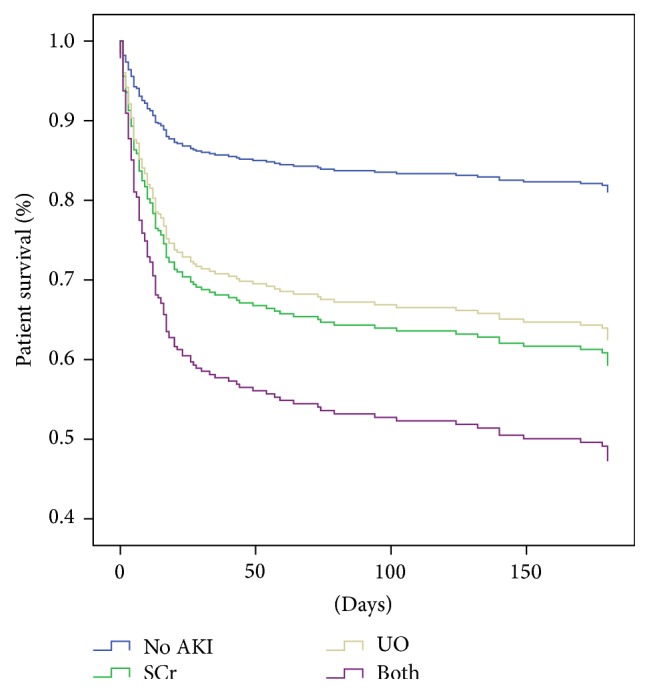
Cox proportional regression analysis curves, classifying patients according to their fulfilled criteria: no AKI and AKI by SCr elevation, UO decrease, or both criteria. Hazard ratios for 6-month mortality were HR 2.24 (95% CI 1.26–4.09) for UO criterion, HR 2.48 (95% CI 1.30–4.76, *p* = 0.006) for SCr criterion, and HR 3.56 (95% CI 2.03–6.24, *p* < 0.001) for both criteria.

Patients with a diagnosis of AKI met only by UO had a similar risk of death to those who had elevated SCr without oliguria as follows: UO with HR 2.24 (95% CI 1.26–4.09, *p* = 0.009) and SCr with HR 2.48 (95% CI 1.30–4.76, *p* = 0.006). Patients who developed both criteria had a greater risk of death, HR 3.56, 95% CI 2.03–6.24, *p* < 0.001 (Figure 2).  Table 2 shows the multivariate models.

### 3.4. Renal Function at Six Months

Five patients received RRT during their ICU stay, two recovered their renal function, and three died.

Two hundred and thirty-two patients survived at 6 months, of whom 177 had a measurement of creatinine, and 87.6% of these patients had an eGFR higher than 60 mL/min/1.73 m^2^. During the follow-up, one patient started RRT, and two refused to start RRT and received palliative care.

## 4. Discussion

We found that 69.4% of our patients developed some degree of AKI during their ICU stay, which is higher than reported in noncancer patients [2, 3]. The higher number of cases with AKI is consistent with the increased risk of renal injury previously described in cancer patients [7, 8]. Moreover, the use of new criteria proposed for diagnosis and classification helped to identify a greater number of cases of AKI, as demonstrated in critically ill hematological patients whose incidence of AKI is 66.5% [15].

According to our hypothesis, the development of AKI is associated with an increased mortality at six months. This increase is progressive and evident even with smaller increases in serum creatinine.

Studies frequently do not report urine output and only consider the value of serum creatinine to diagnose acute kidney injury and classify its severity. Our data showed that all AKI stages defined by SCr criteria were associated with mortality, but considering the UO criteria, we might be able to detect more AKI patients.

When we classified AKI by urine output, the presence of AKI stage 1 was not an independent factor for mortality in the multivariate analysis, but stages 2 and 3 were independently associated with the six-month mortality. This finding occurs because, initially, oliguria is a physiological response to decreased intravascular volume [1]. However, if oliguria lasts more than 12 hours, it is related to renal tubular dysfunction and might result in fluid overload [21–23].

Considering the worst AKI grade, reached by either SCr or UO, all three grades were independently and progressively associated with higher mortality. Furthermore, stage 2 and stage 3 have a higher risk of requiring mechanical ventilation. According to this finding, previous studies suggest evaluating the UO to avoid underestimating the incidence and grade of AKI potentially delaying diagnosis and interventions [24–26].

Patients meeting at least one AKI criterion had higher mortality rates than those who did not develop AKI, even those patients with isolated oliguria without SCr elevation, which was more frequent than isolated creatinine elevation without oliguria. Isolated oliguria might be the only sign of AKI because volume overload could dilute serum creatinine and avoid its elevation, and oliguria could be the only detectable AKI sign [25–27]. Consequently, patients meeting both SCr and UO criteria have even worse outcomes because this finding represents severe renal impairment.

Most of our patients who survived at six months had an acceptable renal function, with an eGFR greater than 60 mL/min/1.73 m^2^; therefore, renal impairment was not a contraindication to receiving appropriate chemotherapy.

Previous studies suggested that CICP patients with AKI had a higher mortality compared to noncancer patients [10, 12]. Nevertheless, studies published in recent years have demonstrated similar mortality rates to noncancer ICU patients. The better survival results are attributed to advances in ICU care and better access in this population to critical care [15, 28, 29].

In addition to AKI stages, the most important prognostic factor in our population was the use of mechanical ventilation. This finding was consistent in all multivariate models.

Previous studies have reported that mechanically ventilated cancer patients have a worse prognosis than noncancer critically ill patients [30]. Other variables independently associated with mortality were leukopenia and malignancy extension, which might relate to the fact that most of our patients with leukopenia had hematological malignancies or were septic.

### 4.1. Limitations

Our study has several limitations. First, we have a selection bias, because patients admitted to our ICU have a possibility of cancer control or are admitted during the assessment of their cancer status. Second, given the observational nature of the study, there were no uniform criteria for starting RRT, and few patients received hemodialysis, mainly because of end of life decisions. Moreover, the majority of our patients already had AKI at the time of ICU admission, so it is not possible to determine the predisposing factors. Despite these limitations, AKI in cancer patients remains an area of uncertainty, and most of our results are comparable to those reported in larger series including noncancer ICU patients.

## 5. Conclusion

Almost 70% of CICP developed AKI when evaluated by using the KDIGO SCr and UO diagnostic criteria. All AKI stages suggest a progressively greater mortality risk independently of cancer-associated factors. Urinary output surveillance might increase the sensitivity of detecting AKI patients in the ICU.

## Figures and Tables

**Table 1:** Patients classified by maximum AKI stage (SCr and UO).

Variables	Total *n* = 389	No AKI *n* = 119	AKI grade 1 *n* = 101	AKI grade 2 *n* = 84	AKI grade 3 *n* = 85	*p* value^*∗*^
Characteristics at ICU admission						
Male gender, *n* (%)	180 (46.3)	50 (42.0)	46 (45.5)	39 (46.4)	45 (52.9)	0.492
Age (years), median (IQR)	50 (35–61)	43 (30–54)	51 (42–61)	55 (39–68)	55 (36–66)	0.001
BMI (kg/m^2^), median (IQR)	26 (22.7–29.1)	25.3 (22.4–27.7)	26.1 (22.2–29.3)	27.6 (24.5–30.8)	25.7 (22.1–28.6)	<0.001
Hospital days prior to ICU, median (IQR)	2 (1–5)	1 (1–3)	2 (1–7)	2 (1–8)	2 (1–6)	0.012
APACHE II score, median (IQR)	15 (11–20)	12 (9–16)	13 (10–18)	17 (13–21)	22 (17–27)	<0.001
SOFA at admission, median (IQR)	6 (3–9)	3 (2–6)	5 (3–8)	8 (5–9)	10 (7–13)	<0.001
Sepsis, *n* (%)	121 (31.1)	22 (18.5)	26 (25.7)	30 (35.7)	43 (50.6)	<0.001
Leukopenia, *n* (%)	72 (18.5)	16 (13.4)	11 (10.9)	20 (23.8)	25 (29.4)	0.003
Total bilirubin, median (IQR)	1 (0.7–1.6)	0.9 (0.6–1.5)	0.9 (0.5–1.5)	1 (0.7–1.7)	1.1 (0.8–1.95)	0.031
Lactate, median (IQR)	2.0 (0.7–1.6)	1.6 (1.2–2.8)	1.9 (1.1–3.4)	2.2 (1.4–3.7)	3.4 (1.7–6.4)	<0.001
Malignancy						
Hematologic malignancy, *n* (%)	109 (28)	21 (17.6)	23 (22.8)	32 (38.1)	33 (38.8)	<0.001
Solid tumor, *n* (%)	280 (72)	98 (82.4)	78 (77.8)	52 (61–9)	52 (61.2)	
Hematologic extension						
Local *n* (%)	78 (20)	12 (10)	20 (19)	23 (27.4)	23 (27)	0.181
Disseminated *n* (%)	31 (8.0)	9 (7.6)	3 (2.9)	9 (10.7)	10 (11.8)	
Solid tumor extension						
Local *n* (%)	157 (40.4)	59 (49.6)	44 (43.6)	32 (38)	22 (25.9)	0.150
Metastatic *n* (%)	123 (31.6)	39 (32.8)	34 (33.7)	20 (23.8)	30 (35.3)	
Comorbidities						
Diabetes mellitus, *n* (%)	77 (19.8)	12 (10.1)	22 (21.8)	24 (28.6)	19 (22.4)	0.008
Hypertension, *n* (%)	85 (21.9)	21 (17.6)	22 (21.8)	22 (26.2)	20 (23.5)	0.513
HIV, *n* (%)	11 (2.8)	1 (0.8)	3 (3.0)	2 (2.4)	5 (5.9)	0.217
Congestive heart failure, *n* (%)	22 (5.7)	4 (3.4)	5 (5)	9 (10.7)	4 (4.7)	0.180
Previous myocardial infarct, *n* (%)	29 (7.5)	6 (5)	7 (6.9)	8 (9.5)	8 (9.4)	0.536
Chronic liver disease, *n* (%)	26 (6.7)	10 (8.4)	6 (5.9)	4 (4.8)	6 (7.1)	0.776
Previous renal function						
Baseline creatinine, median (IQR)	0.8 (0.6–0.9)	0.7 (0.6–0.88)	0.8 (0.66–0.93)	0.7 (0.6–0.99)	0.8 (0.58–1.05)	0.037
Baseline eGFR, median (IQR)	100 (80–117)	107 (89–124)	97 (79–110)	98 (79–116)	98 (70–121)	0.002
Chronic renal failure, *n* (%)	29 (7.5)	3 (2.5)	5 (5)	10 (11.9)	11 (12.9)	0.008
ICU hospitalization						
Mechanical ventilation, *n* (%)	248 (63.8)	46 (38.7)	74 (73.3)	62 (73.8)	66 (77.6)	<0.001
Vasopressors, *n* (%)	231 (59.4)	39 (32.8)	60 (59.4)	61 (72.6)	71 (83.5)	<0.001
Fluid balance (liters), median (IQR)	3.8 (0.9–8.7)	1.0 (0–2.3)	4.3 (0.9–7.8)	6.6 (3.5–10.7)	9.0 (4.3–12.6)	<0.001
Outcome						
ICU stay (days), median (IQR)	4 (2–7)	2 (2–4)	5 (3–10)	6 (4–9)	5 (3–9)	<0.001
Death at ICU, *n* (%)	87 (22.4)	6 (5)	13 (12.9)	22 (26.2)	46 (54.1)	<0.001
Death at 180 days, *n* (%)	157 (40.4)	18 (15.1)	37 (36.6)	43 (51.2)	59 (69.4)	<0.001

AKI: acute kidney injury; ICU: intensive care unit; IQR: interquartile range; SD: standard deviation; BMI: body mass index; APACHE: Acute Physiology on Chronic Health Evaluation; SOFA: Sequential Organ Failure Assessment; HIV: human immunodeficiency virus; eGFR: estimated glomerular filtration rate; SCr: serum creatinine; UO: urinary output. ^*∗*^We performed comparisons between AKI stages classified by worst AKI by either SCr or UO.

**Table 2:** Multivariate Cox-regression analyses for factors associated with 6-month mortality by using different AKI criteria.

	Model 1: SCr alone			Model 2: UO alone			Model 3: maximum grade by SCr or UO			Model 4: SCr, OU, or both criteria			
	HR	95% CI	*p* value	HR	95% CI	*p* value	HR	95% CI	*p* value		HR	95% CI	*p* value
Characteristics at ICU admission													
Male gender	1.23	0.87–1.74	0.243	1.18	0.84–1.67	0.339	1.21	0.86–1.72	0.278		1.18	0.83–1.69	0.350
Age (years)	1.00	0.99–1.01	0.881	1.00	0.98–1.01	0.334	1.00	0.99–1.01	0.582		1.00	0.99–1.01	0.668
Hospital days prior to ICU	1.00	0.98–1.02	0.825	1.00	0.98–1.02	0.768	1.00	0.98–1.02	0.973		1.00	0.98–1.02	0.946
Sepsis	1.21	0.85–1.71	0.295	1.27	0.90–1.79	0.171	1.16	0.82–1.64	0.403		1.20	0.85–1.71	0.299
Leukopenia	1.88	1.22–2.88	0.004	1.85	1.22–2.82	0.004	1.78	1.16–2.74	0.009		1.87	1.22–2.87	0.004
Malignancy													
Hematologic malignancy	1.47	0.98–2.22	0.067	1.50	1.01–2.24	0.045	1.42	0.94–2.14	0.095		1.52	1.01–2.30	0.047
Neoplasm extension	1.62	1.16–2.25	0.004	1.64	1.18–2.27	0.003	1.59	1.14–2.20	0.006		1.66	1.20–2.30	0.002
ECOG	1.18	1.02–1.37	0.027	1.15	0.99–1.34	0.073	1.15	0.99–1.33	0.060		1.17	1.01–1.36	0.037
ICU hospitalization													
Mechanical ventilation	2.60	1.66–4.07	<0.001	2.30	1.43–3.67	<0.001	2.38	1.52–3.72	<0.001		2.14	1.35–3.40	<0.001
Vasopressor	1.36	0.89–2.07	0.162	1.45	0.96–2.19	0.081	1.28	0.85–1.94	0.237		1.33	0.88–2.01	0.173
Acute kidney injury													
No AKI		*Ref*	*Ref*		*Ref*	*Ref*		*Ref*	*Ref*	No AKI		*Ref*	*Ref*
Stage 1 AKI	1.70	1.07–2.70	0.025	1.34	0.83–2.15	0.230	2.04	1.14–3.68	0.017	SC	2.48	1.30–4.76	0.006
Stage 2 AKI	1.98	1.22–3.21	0.006	2.19	1.36–3.54	0.001	2.73	1.53–4.88	0.001	UO	2.24	1.26–4.09	0.009
Stage 3 AKI	2.33	1.50–3.61	<0.001	4.18	2.52–6.92	<0.001	4.50	2.52–8.02	<0.001	Both^*∗*^	3.56	2.03–6.24	<0.001

AKI: acute kidney injury; SCr: serum creatinine; UO: urinary output.

^*∗*^Both Refer to patients who fulfilled SCr elevation and UO criteria.
